# Sexual dimorphism in bladder cancer: a review of etiology, biology, diagnosis, and outcomes

**DOI:** 10.3389/fphar.2023.1326627

**Published:** 2024-01-12

**Authors:** Sheng Zhu, Huasheng Zhao

**Affiliations:** ^1^ Department of Urology, Guilin Hospital of the Second Xiangya Hospital, Central South University, Guilin, China; ^2^ Department of Urology, ShaoYang Hosptial, Affiliated to University of South China, ShaoYang, China

**Keywords:** bladder carcinoma, etiology, biology, diagnosis, sexual dimorphism

## Abstract

Bladder carcinoma represents a prevalent malignancy, wherein the influence of sex extends across its incidence, biological attributes, and clinical outcomes. This scholarly exposition meticulously examines pertinent investigations, elucidating the nuanced impact of sex on bladder cancer, and posits cogent avenues for future research and intervention modalities. In the initial discourse, an exhaustive scrutiny is undertaken of the etiological underpinnings of bladder cancer, encompassing variables such as tobacco consumption, occupational exposures, and genetic aberrations. Subsequently, a comprehensive dissection unfolds, delving into the intricate biological disparities inherent in sex vis-à-vis the initiation and progression of bladder cancer. This analytical framework embraces multifaceted considerations, spanning sex hormones, sex chromosomal dynamics, metabolic enzymatic cascades, and the intricate interplay with the microbiome. Lastly, a synthesized exposition encapsulates the ramifications of gender differentials on the diagnostic and prognostic landscapes of bladder cancer, underscoring the imperative for intensified investigative endeavors directed towards elucidating gender-specific variances and the formulation of tailored therapeutic strategies.

## 1 Introduction

**FIGURE 1:**
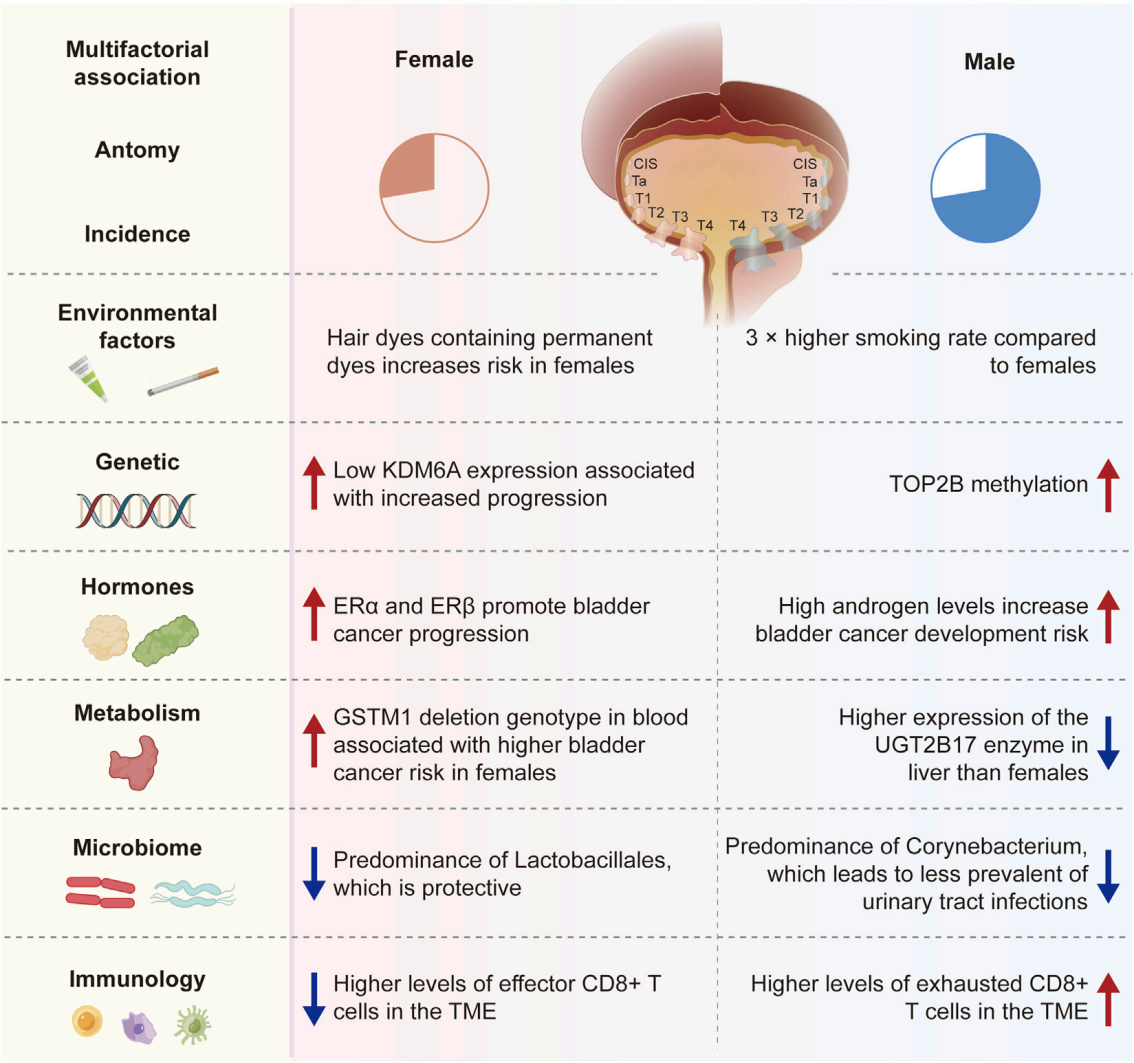
The connection between sex and bladder cancer in detail from four distinct aspects: etiology, biology, diagnosis, and outcomes.

One prevalent malignant tumor is bladder cancer (BC) (Richters et al., 2020). It has been widely reported that there are gender disparities in bladder cancer patients’ epidemiology, diagnosis, and prognosis. Bladder cancer is more common in men than in women worldwide but a diagnosis of advanced bladder cancer is more common in females (Scosyrev et al., 2009). Extensive research has confirmed women’s association with poorer oncological outcomes, including an elevated likelihood of mortality, disease recurrence and disease progression (Zeegers et al., 2000; Castelao et al., 2001; Boffetta, 2008). Treatment disparities cannot fully explain these differences in survival rates between genders. Currently, various hypotheses, including physiological anatomical structures, disease phenotypes, hormone changes, sex epigenomics, diagnostic delays, and treatment strategies, are being used to explain sex-specific adverse outcomes. Therefore, this article will explore the connection between sex and bladder cancer in detail from four distinct aspects: etiology, biology, diagnosis, and outcomes (Figure 1).

## 2 Etiological difference in bladder cancer

### 2.1 Smoking

Smoking is widely recognized as one of the most significant risk factors for bladder cancer, as those who smoke have a notably higher chance of getting the disease than people who do not smoke (Strope and Montie, 2008). Smoking is linked to bladder cancer in one-third of women and at least 50% of men (Zeegers et al., 2000). Compared to female smokers, male smokers have a higher risk of developing bladder cancer (Castelao et al., 2001), which may be attributed to higher smoking rates and metabolic differences in men. Furthermore, studies have shown a linear correlation between smoking and the risk of bladder cancer, and quitting smoking can reduce the incidence of bladder cancer (Boffetta, 2008). In men and women, the incidence of bladder cancer is roughly 4:1 when smoking intensity is similar (Krabbe et al., 2015). The main cause of the gender difference in bladder cancer incidence is generally considered to be smoking. However, according to the study, there is a 3.31 relative risk of incidence of bladder cancer in women when smoking rates are 70% for males and 10% for women, which is lower than the global average of 4.04. This implies that the variations in bladder cancer incidence across genders can only be partially explained by smoking (Hemelt et al., 2009).

### 2.2 Occupations

Chemical substances and carcinogens in certain occupational environments have different effects on the risk of bladder cancer in women and men, which may be related to gender differences in metabolic pathways and hormone levels. For example, occupations including driving, rubber manufacturing, hair styling, and petroleum product processing can expose workers to higher levels of aromatic amine chemicals, raising their risk of bladder cancer (Reulen et al., 2008; Samanic et al., 2008; Bevan et al., 2012). Additionally, studies have reported a higher risk of bladder cancer in women who use hair dyes containing permanent dyes, with women with a higher risk for the N-acetyltransferase-2 slow acetylation phenotype (Koutros et al., 2011). The statement indicating a higher risk of bladder cancer in women using hair dyes, especially those containing permanent dyes, in association with the N-acetyltransferase-2 (NAT2) slow acetylation phenotype (Gago-Dominguez et al., 2001; Zhang et al., 2020). However, the evidence is not entirely consistent, and the relationship may be influenced by factors like dose, duration of exposure, and individual susceptibility. Long-term and frequent use of hair dyes has been suggested to be associated with an increased risk of bladder cancer, but findings across studies vary. To better understand the potential risks, assessing blood levels of carcinogenic compounds resulting from hair dye use is crucial, involving biomarkers of exposure or specific metabolite measurements. While references to such blood level measurements may require targeted literature searches, consulting systematic reviews or meta-analyses could offer a broader overview of the available evidence on the association between hair dye use, bladder cancer risk, and the role of dose, duration, and biomarkers. However, in terms of gender disparities, there is a lack of thorough research to assess the connection between bladder cancer and occupation.

### 2.3 Gene mutation

Some gene mutations associated with bladder cancer exhibit different frequencies and effects in men and women. Bladder cancer is typically believed to occur due to the entry of chemical substances in tobacco into the bloodstream, accumulating in the urine through filtration by the kidneys, and subsequently causing mutations in bladder cell genes. However, gene mutations are random events that may be influenced by the cellular microenvironment and can occur even without external stimuli (Wang et al., 2021; Lin et al., 2020). HRA, KRAS2, RB1, and FGFR3 are known somatic mutation genes associated with bladder cancer (Kiemeney et al., 1997; Cappellen et al., 1999). Gene changes such as STAG2, TERT, ESPL1, UTX, MLL, MLL3, CREBBP, EP300, NCOR1, and ARID1A are also linked to bladder cancer (Fliss et al., 2000; Solomon et al., 2013; Cha and Bochner, 2015). A higher risk of bladder cancer is associated with PTEN mutations in persons with breast cancer and thyroid cancer (Hemelt et al., 2009; Cordes et al., 2013). Studies have identified differences in gene mutation patterns between different genders in bladder cancer, particularly X chromosome-based genes (Gul et al., 2021). In a large clinical cohort study, 58 genes were found to undergo significant mutations in patients with muscle-invasive bladder cancer (MIBC), clustering into five subtypes (Cumberbatch and Catto, 2018). The basal-squamous subtype was more common in women. This suggests that next-generation sequencing technologies can provide a more comprehensive data foundation for exploring bladder cancer, which is of great significance for understanding the mechanisms of bladder cancer development and the reasons behind gender differences.

## 3 Biological differences in bladder cancer

### 3.1 Sex chromosome and epigenetics

Research has indicated that the loss of the Y chromosome in males raises the risk of cancer. In contrast, females with Turner syndrome (loss or partial loss of the X chromosome) have a significantly increased risk of bladder cancer, while males with Klinefelter syndrome (extra copy of the X chromosome) have a significantly reduced risk of solid tumors (Ji et al., 2016; Theodorescu et al., 2022). Findings further show that sex hormones do not influence gender-biased effects of the X chromosome and that an extra copy of the X chromosome guards against bladder cancer (Kaneko and Li, 2018). Lysine-specific demethylase 6A is one of the most commonly mutated genes in bladder cancer (KDM6A), a tumor suppressor found on the X chromosome (Xp11.3) (Robertson et al., 2018; Koti et al., 2020). As a demethylase for trimethylation of histone H3 at lysine 27 (H3K27me3), KDM6A mutations result in the availability of H3K27 for acetylation. H3K27me3 modification is a transcriptionally repressive epigenetic mark that can form bivalent domains with the active transcription mark H3K4me3, keeping genes poised (Voigt et al., 2013). Additionally, studies have demonstrated the involvement of KDM6A in mediating the methyltransferase activity of H3K4me1 (Jang et al., 2017; Rickels et al., 2017). Cohort analysis has shown an association between reduced KDM6A expression and female bladder cancer progression (Kaneko and Li, 2018). The UTY (KDM6C) gene on the Y chromosome is a homologous gene of KDM6A (Lan et al., 2007). UTY can compensate for KDM6A mutation or deletion on the X chromosome on the Y chromosome (Lam et al., 2022). According to studies, deleting compensatory UTY on the Y chromosome may increase men’s bladder cancer risk (Forsberg et al., 2014).

The interaction between sex hormones and their corresponding receptors plays a crucial role in the occurrence and development of bladder cancer, and the differences in hormone levels between genders may explain the variations in bladder cancer incidence. Research has shown that the presence of the androgen receptor (AR) gene, which is situated on the X chromosome (Xq11-12), could potentially explain the differences in bladder cancer occurrence across genders. Bladder cancer occurrence and development can be facilitated by AR mutations that affect ligand binding (Rahmani et al., 2013; Izumi et al., 2014a). According to multiple studies, androgens stimulate bladder cancer growth via classical and non-classical AR pathways (Izumi et al., 2014a; Deng et al., 2021). Based on the data, reducing bladder cancer invasion may be achieved by inhibiting AR, and anti-androgen Enzalutamide can reduce bladder cancer cell invasion (Deng et al., 2021). Targeting AR can also lower the expression of CD44, a gene linked to the invasion behavior of bladder tumors (Sottnik et al., 2021). Boorjian et al. have shown that more invasive tumor stage and AR expression are negatively correlated in bladder cancer, with lower levels of AR expression in female patients (Boorjian et al., 2004). However, several studies have not detected significant differences in tumor AR expression between genders (Mir et al., 2011; Tuygun et al., 2011; Mashhadi et al., 2014). A large body of research has shown that androgens and their downstream signaling pathways may not only be related to tumor progression in muscle-invasive bladder cancer (MIBC) but also have the potential to become therapeutic targets (Gakis and Stenzl, 2013; Xu et al., 2013; Izumi et al., 2014b; Mashhadi et al., 2014).

Estrogen binds to one of the two nuclear receptors, ERα and ERβ, structurally and functionally distinct. It has been found that ERα inhibits bladder cancer from occurrence, while ERβ has been shown to promote its development (Hsu et al., 2013)。However, both ER subtypes have been found to promote bladder cancer progression. Both nuclear estrogen receptors (ERα and ERβ) are responsible for transducing hormone signals into transcriptional responses (Xu et al., 2013). Shen et al. detected that ERβ is the predominant subtype expressed in UCB and that high levels of ERβ expression correlate with higher tumor grades (Shen et al., 2006; Tuygun et al., 2011). The team has also shown that exogenous estrogens promote bladder cancer cell growth *in vitro*, which can be inhibited by anti-estrogen drugs such as raloxifene (Shen et al., 2006). However, other studies have found that menopause raises the risk of bladder cancer (McGrath et al., 2006), and the use of combined estrogen and progesterone therapy can reduce this risk, which is not associated with estrogen use alone (Daugherty et al., 2013).

**FIGURE 2:**
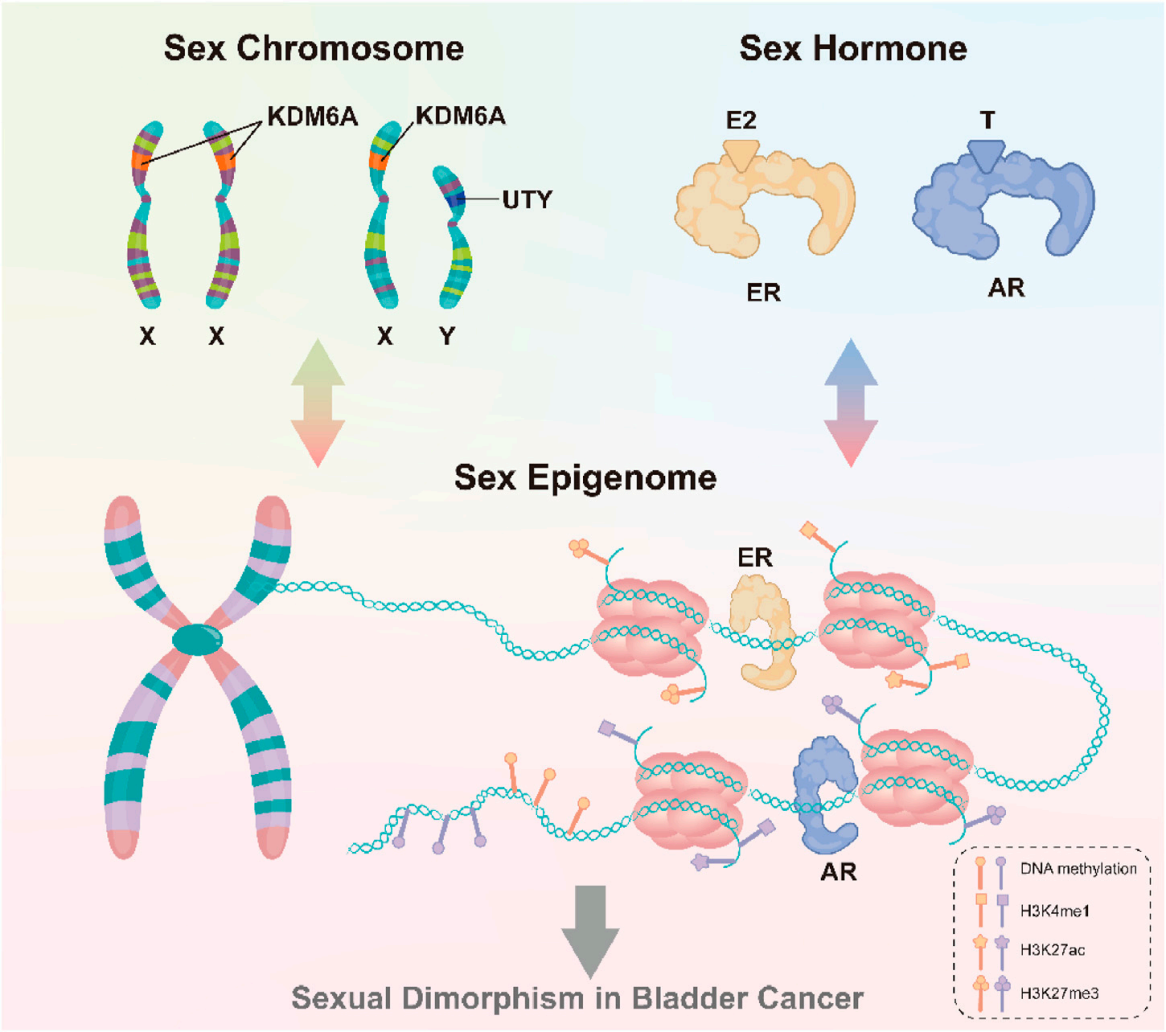
Sex chromosomes and sex hormones, through the sex epigenome, collectively influence gene expression and response to the environment, thus leading to the observed gender differences in bladder cancer.

In summary, differences in bladder cancer incidence rates across genders are partly explained by the interaction of sex hormones and sex chromosomes (Figure 2). One type of cancer that has been connected to sex steroid hormones and the receptors on the surface of cells that they bind to is bladder cancer (Gakis and Stenzl, 2013; Xu et al., 2013), future research directions could focus on molecular mechanisms underlying gender-related incidence rate differences and develop potential therapies for bladder cancer targeting the androgen-AR signaling pathway or identifying patient populations that may benefit the most from preventive treatments.

### 3.2 Metabolic enzymes

Gender differences in metabolic detoxification may contribute to the varying incidence rates of bladder cancer (Zhang, 2013). The ability of the liver pathway to degrade carcinogens differs between genders, leading to varying degrees of carcinogen accumulation in the urothelium (Buckley and Klaassen, 2007; Zhang, 2013). UDP-glucuronosyltransferases (UGTs), which are involved in the liver’s process for breaking down aromatic amines, are responsible for eliminating exogenous and endogenous substances (Zhang, 2013; Hu et al., 2016; Meech et al., 2019), smoke from cigarettes contain carcinogens called aromatic amines that damage DNA. Therefore, the development of bladder cancer is significantly influenced by the detoxification of aromatic amines. Studies showed a noteworthy reduction in the UGT1A subtype enzyme expression in elevated bladder cancer compared to normal urothelium (Izumi et al., 2013). In liver tissue, males have higher expression of the UGT2B17 enzyme than females (Gallagher et al., 2010), indicating differences in enzyme activity between genders in metabolizing carcinogens. Additionally, it has been found that androgen receptor-mediated signaling inhibits UGT expression in bladder (Izumi et al., 2013; Zhang, 2013) and prostate cancer (Takayama et al., 2007), indicating a gender bias in the UGT detoxification pathway in bladder cancer. While several research studies have shown a connection between UGT and bladder cancer, there is still a need for large clinical cohort studies to definitively establish the significance of UGT in gender differences in bladder cancer (Hu et al., 2016).

Moreover, glutathione-S-transferase M1 (GSTM1), which binds to reduced glutathione to catalyze the detoxification of foreign substances, is also considered a metabolic target for gender differences in bladder cancer incidence rates (Hengstler et al., 1998; Karagas et al., 2005; Yu et al., 2017). GST activity regulates exposure to carcinogens in the bladder urothelium and affects bladder cancer risk. A study found that females with the GSTM1 deletion genotype were more likely to develop bladder cancer, although this link was not found in males (Karagas et al., 2005; Salinas-Sánchez et al., 2011). Additionally, female smokers were shown to have a higher risk of bladder cancer when their blood samples had the GSTM1 deletion genotype, but not in non-smokers (Karagas et al., 2005), possibly due to females with the GSTM1 null genotype being unable to metabolize carcinogens in cigarette smoke. Additional clinical data is required to verify the relationship between GSTM1 and gender differences in bladder cancer.

According to the research findings on the comprehensive regulation of androgens on cell metabolism and detoxification, it can be hypothesized that the reason for the predominance of males in bladder cancer may be due to gender differences in enzymes responsible for carcinogen metabolism, resulting in varying degrees of exposure to environmental carcinogens (such as carcinogens in cigarette smoke), ultimately leading to differences in incidence rates. However, further research is needed to explore other potential metabolic targets contributing to differences in carcinogen processing and to conduct larger clinical cohort studies to determine the relationship between the expression of gender-related metabolic enzymes and the biology of bladder cancer.

### 3.3 Microbiome and microbiota

Data show that the microbiota may be associated with over one-fifth of malignant tumors (Garrett, 2015). The mechanisms of interaction between the microbiota and human cells involve at least one of the following: direct impacts on the host’s innate immune system, interactions with their biochemistry, and host cell proliferation and death (Bhatt et al., 2017). The development and progression of bladder cancer involve a multifaceted interplay of sex hormones, chromosomes, liver enzymes, and the microbiome. Sex hormones, including estrogen and androgens, exert influence through receptors expressed in bladder cells, with androgens stimulating cancer growth. Alterations in sex chromosomes, such as Y chromosome loss in males and X chromosome anomalies in females, contribute to varying risks. Liver enzymes, notably metabolic detoxification enzymes like UDP-glucuronosyltransferases (UGTs) and glutathione-S-transferase M1 (GSTM1), exhibit gender-specific differences, affecting carcinogen detoxification and thus bladder cancer risk. The urinary microbiota, with variations between genders, has been linked to bladder cancer progression, suggesting that distinct microorganisms may create different local environments influencing tumor establishment. Understanding these factors in diverse pathological conditions, such as muscle-invasive or non-urothelial bladder cancer, is pivotal for personalized diagnostic and therapeutic strategies. Further research is essential to elucidate the nuanced mechanisms and interactions shaping bladder cancer development under different pathological contexts.

For example, *Salmonella typhi* may activate the Wnt/β-catenin pathway, which could lead to hepatobiliary and colorectal cancer (Villaseñor et al., 2017). As urine can collect many disease-related changes, it can serve as a good source of biomarkers. According to recent research, bladder cancer and changes in the urine microbiome are related (Alfano et al., 2016; Bučević Popović et al., 2018). Because of the physiological variations between males and females, females are more prone to urinary tract infections. Wu et al. found that individuals with bladder cancer had a markedly elevated urinary microbiota bacterial abundance, with reductions in *Serratia*, *Proteus*, and Roseomonas and increases in *Acinetobacter*, Anaerococcus, and Sphingobacterium (Wu et al., 2018). A series of studies have also demonstrated that dysbiosis of the urinary microbiota may influence bladder cancer progression (Bi et al., 2019; Mai et al., 2019). These investigations suggest that distinct microorganisms in the urine of men and women may produce comparatively different local habitats, which may encourage or prevent the establishment of bladder tumors. Interactions between the immune system’s sex hormones and the urine microbiome may be closely related (Curtiss et al., 2017). Sequencing results of urine samples showed that Lactobacillales and Corynebacterium dominate the urinary microbiota in females and males, respectively (Fouts et al., 2012). Lactobacillales have a protective effect against urinary tract infections (Kim and Park, 2018), and clinical trials have shown that oral administration of *Lactobacillus* preparations can slow down bladder cancer recurrence (Aso et al., 1995). Corynebacterium can influence the composition of the microbiota by hydrolyzing lipids and releasing free fatty acids with antimicrobial activity (Chen et al., 2018). Pederzoli et al. found a higher level of *Klebsiella* species in the urine of women with bladder cancer than in women with good health (Pederzoli et al., 2020). *Klebsiella* is linked to the development of bladder cancer (Mai et al., 2019), possibly due to the release of toxins by *Klebsiella* that cause DNA damage (Kaur et al., 2018). The aforementioned experimental results demonstrate that differences in urinary microbiota between different genders can be one of the reasons explaining the differences between biological genders in bladder cancer. The relationship between microbiota and cancer has been explained by various postulated processes, such as the induction of chronic inflammation, the promotion of cell proliferation, and the activation of procarcinogens (Xu et al., 2014). However, large-scale studies are still needed to clarify the precise connection between differences in the distribution of microbiota and the development of bladder cancer.

### 3.4 Immunobiology

Gender differences in immune responses may also impact the development and prognosis of bladder cancer. In patients with advanced bladder cancer, the microbiota’s modulation affects how well they respond to systemic and adjuvant *Bacillus* Calmette-Guérin (BCG) immunotherapy (Killock, 2018; Routy et al., 2018; Stenehjem et al., 2018; Zitvogel et al., 2018). Studies suggest that *Lactobacillus* iners, which dominate the urinary microbiota in females, may preferentially bind to fibronectin, competing with BCG and weakening its efficacy (McMillan et al., 2013). When platinum-based chemotherapy fails to treat advanced bladder cancer patients, or they are not eligible for it, immune checkpoint inhibitors become the standard treatment. Studies suggest that the gut microbiota composition may affect how well immune checkpoint blockade drugs work for metastatic melanoma (Matson et al., 2018). However, there is currently no research involving bladder cancer patients with metastases. A recent study found an association between Y chromosome loss and poorer prognosis (Abdel-Hafiz et al., 2023). Y chromosome loss in bladder cancer refers to the condition where cells, typically in males, experience a partial or complete loss of the Y chromosome. This genetic alteration is associated with more aggressive tumor characteristics, including increased invasiveness and the development of an immunosuppressive tumor microenvironment. Despite its negative impact on cancer aggressiveness, bladder cancers with Y chromosome loss paradoxically exhibit heightened sensitivity to immune checkpoint blockade therapy, suggesting a potential therapeutic vulnerability. The presence of Y chromosome loss in bladder cancer patients could serve as a prognostic marker, guiding clinical decision-making and personalized treatment strategies. Bladder cancers with Y chromosome loss exhibit more invasive and immunosuppressive tumor microenvironments but are also more sensitive to immune therapy. This study demonstrates an association between Y chromosome loss and increased response to immune checkpoint blockade therapy in humans and mice, suggesting a potential therapeutic avenue for this subset of bladder cancer. Additionally, data show a gender bias effect of CD8^+^ T cells, leading to faster male tumor growth (Kwon et al., 2020). The study further indicates an increase in effector CD8^+^ T cells in the tumor microenvironment (TME) of females, while males have higher levels of exhausted CD8^+^ T cells in the TME (Kwon et al., 2020).

## 4 Sex-specific diagnostic differences

While males are more likely to have BC, females are frequently detected at an advanced stage (Mungan et al., 2000a; Lotan et al., 2005; Scosyrev et al., 2009; Fajkovic et al., 2011; Mallin et al., 2011; Rink et al., 2012; Kluth et al., 2014; Mitra et al., 2014; Soave et al., 2015). Over 23,000 MIBC patients were included in a study by Marieke J. Krimphove et al. (Fickenscher, 1999), which revealed that the proportion of female patients with non-urothelial bladder cancer was significantly higher (15.1% in females vs. 9.9% in males *p* < 0.001). Among patients with histological variations, females exhibited poorer pathological features at diagnosis, with a higher prevalence of squamous cell carcinoma (46.9% in females vs. 28.7% in males; *p* < 0.001), while males had a higher prevalence of neuroendocrine carcinoma (12.3% in females vs. 21.8% in males; *p* < 0.001) or micropapillary differentiation carcinoma (3.8% in females vs. 9.0% in males; *p* < 0.001). Although biological differences may contribute to this phenomenon, the timeliness and quality of the initial evaluation of hematuria, the most prevalent presenting symptom in both genders, maybe the main reason for the diagnostic stage differences (Fickenscher, 1999; Shephard et al., 2012). Several studies have demonstrated that compared to men, women with hematuria are less likely to see urologists for evaluation (Johnson et al., 2008; Nieder et al., 2010; Henning et al., 2013). Johnson et al. (Nieder et al., 2010) observed a 65% higher referral rate of urologists for male patients experiencing recurrent hematuria compared to their female counterparts (median follow-up time of 26.5 months: 47% vs. 28%; *p* < 0.001). Henning et al. (Henning et al., 2013) conducted a survey of 168 UCB patients (including 38 female patients) and found no gender differences in the initial symptoms (*p* > 0.05). However, 78% of male patients directly consulted urologists, while only 55% of female patients did so (*p* < 0.05). 49.2% of female patients and 19% of male patients were treated symptomatically without receiving a specific diagnosis (*p* < 0.04), which did not result in any significant differences in tumor staging at the time of initial transurethral resection (Henning et al., 2013). Furthermore, a retrospective analysis of blood in urine patients in an institutional electronic medical record database found that only 8% of females were referred to urologists (Buteau et al., 2014). Similarly, an analysis of US and UK populations found that females had a significantly lower likelihood of receiving timely and complete blood in urine evaluation than males (Lyratzopoulos et al., 2013; Garg et al., 2014; Bassett et al., 2015). Between 2000 and 2007, 35,646 people were diagnosed with UCB following blood in urine testing, based on an examination of the Surveillance, Epidemiology, and End Results (SEER) database. The average time from the first appearance of blood in urine to consulting a urologist was 27 days, significantly longer for female patients (Garg et al., 2014). The database comprised 100 health insurance plans from around 40 large US companies. According to the study, the average time it took for females to be diagnosed with bladder cancer was significantly longer than that of males. For example, the average diagnosis time for females was 85.5 days (95% confidence interval 81.3–89.4 days) compared to 73.6 days (71.2–76.1 days) for males (*p* < 0.001). After receiving an initial diagnosis of hematuria, this difference still exists at 3 months, 6 months, and 9 months; females have a 26%, 16%, and 23% higher likelihood of experiencing delay, respectively. Additionally, a delay of more than 6 months between the onset of hematuria and diagnosis of bladder cancer is more common in females (17.3% vs. 14.1% in males; *p* < 0.001).

Most bladder cancers originate in the urothelium, the bladder cavity’s lining epithelium. Since the early signs and symptoms resemble urinary tract infections, female patients often receive antibiotic treatment before undergoing comprehensive urological evaluation. For example, Cohn et al. (Cohn et al., 2014) found in their survey that females undergo more urine analysis and urine culture and receive more urinary tract infection diagnoses before being diagnosed with bladder cancer, with a significantly higher proportion of females receiving antibiotics before diagnosis (40.1% in females vs. 35.4% in males; *p* < 0.001), but fewer females undergo bladder imaging. This phenomenon was also observed by Aziz et al. (Aziz et al., 2015) in a smaller study group. In this study, 61.1% of female UCB patients received antibiotic treatment in the 12 months preceding diagnosis, while only 20% of male patients did (*p* = 0.005). Moreover, voiding difficulties and stomach pain are linked to bladder cancer, and reports indicate that females with these complaints are more likely to receive empirical treatment without further evaluation, with 47% of females receiving treatment without further evaluation in the year before diagnosis, compared to only 19% of males (*p* < 0.05) (Henning et al., 2013). According to Hollenbeck et al. (Bergman et al., 2013), patients who initially present with hematuria but are subsequently identified with bladder cancer (CSM) have a significant risk of cancer-specific mortality when their diagnosis is delayed.

These results show notable gender variations in the evaluation of hematuria, which may lead to an imbalance in the prompt identification of UCB. Females are more likely than males to present with lower urinary tract infection-related symptoms or hematuria. This delay could play a significant role in why females have worse survival rates and are more likely than males to suffer advanced-stage disease. Therefore, it is crucial to educate clinicians on standardized, guideline-based diagnostic and management approaches for all hematuria patients, irrespective of gender (Patel et al., 2008; Davis et al., 2012; Bergman et al., 2013)

## 5 Sex-specific differences in outcomes

### 5.1 Gender disparities in bladder cancer

Aside from the noted variations in the detection stage, female patients with bladder cancer also face an elevated likelihood of cancer-specific mortality compared to their male counterparts (Hashibe et al., 2003; Underwood et al., 2006; Tracey et al., 2009). Although males are diagnosed with bladder cancer at a rate approximately three times higher than females, their likelihood of dying from it is only about twice that of females, resulting in a lower CSM-to-incidence ratio for males (Shariat et al., 2010). Furthermore, female patients diagnosed with bladder cancer have a greater reduction in lifespan compared to males (6.5 years for females vs. 3.9 years for males) (Scosyrev et al., 2012). While some of the survival differences can be attributed to the higher incidence of late-stage disease in females, the differences in the presentation stage cannot fully explain the gender-related survival differences in bladder cancer patients, as studies show that female individuals have a lower prognosis across all disease stages. For example, the female patients’ 5-year survival rate at stage I is 93.7%, a decrease of 2.8% compared to males; for stage II, it is 59.6%, a decrease of 5.9% compared to males; for stage III, it is 49.6%, a decrease of 9.2% compared to males; and for stage IV, it is 15.2%, a decrease of 11.9% compared to males (Mungan et al., 2000a). Some registry-based studies focusing on elderly populations have shown that female UCB patients have higher tumor staging and lower survival rates (Mungan et al., 2000a; Mungan et al., 2000b). A study by Kluth et al. (Moschini et al., 2019) used data from the Japanese Kanagawa cancer registry, which included 13,184 primary UCB patients from Japan between 1954 and 2010. After adjusting for patient age, treatment timing, and histological subtype, they found that CSM increased by approximately 40% in females. Another Japanese population-based registry study revealed that at the time of initial diagnosis, women’s cancer staging was higher than men’s, and the prognosis for women with bladder cancer that was localized or locally advanced was poorer than that of males.

Currently, in patients receiving radical cystectomy (RC) for bladder cancer, there is limited data supporting gender differences in survival rates. Some studies have suggested that female gender is an independent risk factor for CSM after RC (85, 112, 113). For example, Kluth et al. (Kluth et al., 2014) conducted a study analyzing 8,102 patients (including 1,605 females) who underwent RC and found that female gender was an independent predictor of bladder CSM (HR 1.17, *p* = 0.004) after adjusting for various factors. Ingmar Wolff and colleagues (Gago-Dominguez et al., 2001) analyzed studies published between 2012 and 2015 that focused on standardizing care for muscle-invasive bladder cancer (MIBC) and analyzing how gender affects prognosis. Among the 8 RC series studies, 5 reported higher CSM rates in female patients (Kiemeney et al., 1997; Cappellen et al., 1999; Fliss et al., 2000; Reulen et al., 2008; Zhang et al., 2020). Two studies specifically found gender-specific prognostic effects in early-stage disease (Kiemeney et al., 1997; Fliss et al., 2000). In these two studies, female patients also had lower survival rates, especially when it came to younger patients (≤55 years and ≤60 years) and those who had lymphovascular invasion (LVI). However, no gender effect on prognosis was seen in two studies focusing on early invasive T1-high-grade UCB and TURB with intravesical treatment (Solomon et al., 2013; Cha and Bochner, 2015). Additionally, females were more likely to undergo RC (odds ratio [OR] 1.39; 95% CI 1.20–1.61) and had fewer complications (*p* < 0.05).

Regarding a separate relationship between gender and survival following RC, there is, however, conflicting evidence. Using demographic, tumor, and therapeutic data to match 414 female and 2,153 male patients, Mitra et al. (Mallin et al., 2011) found no significant gender differences regarding recurrence-free, cancer-specific, and overall survival. After controlling for tumor stage and other factors, a different study including 398 male and 119 female RC patients found no relationship between postoperative survival and gender. Keck et al. (Cordes et al., 2013) conducted a study on patients undergoing adjuvant chemotherapy and found that female patients had higher CSM in a multivariate analysis (HR 2.40, *p* < 0.001). Zhao et al. studied 233 eligible MIBC patients (177 males [76%] and 56 females [24%]) and 105 NMIBC patients (80 males [76.2%] and 25 females [23.8%]). According to this study, patients with bladder cancer who were female had a poorer prognosis than those who were male at particular stages, and obese females with higher BMI had poorer survival, while females with normal weight (BMI <24) had a higher likelihood of recurrence.

When analyzing gender differences in the prognosis of UCB, it is crucial to focus on patients with T4 bladder tumors. Gender-specific anatomical distinctions are present in the pT4 tumor stage, wherein pT4a tumors extend into the vagina or uterus in females and the prostate in males (Moschini et al., 2019). Women with pT4 bladder cancer had worse outcomes than men, according to a large study based on 583 occurrences of the disease among 4,257 patients (Tilki et al., 2010c). In an analysis by Matthias et al. (May et al., 2013), the 5-year cancer-specific survival rate for 245 pT4a UCB patients who did not receive chemotherapy before undergoing RC was 15% for females and 35% for males (*p* = 0.003). Multivariate analysis showed that female patients had a poorer prognosis. Similarly, an analysis by Danielji et al. of 5,625 SEER database-based RC treatment for pT3–pT4 UCB patients also indicated that females had a higher independent risk of CSM compared to males (HR 1.20; *p* = 0.003) (Liberman et al., 2011a). However, analysis of a small sample (n = 128) of pT4 tumor patients did not find a correlation between gender and survival rates (Kaushik et al., 2014), although this result may be due to the small sample size. Additionally, the results of these reports may be influenced by the heterogeneity of pT4 staging, especially in males. For example, the transmural infiltration of the primary bladder tumor is not considered when ductal and stromal invasion are categorized as pT4a. It is also possible for tumors designated as UCB to be poorly differentiated prostate cancer in cases of extremely undifferentiated tumors (Downes et al., 2013).

The relationship between gender and prognosis differences in NMIBC is also limited and sometimes contradictory. Fabiano et al. (Santos et al., 2015) found that similar to delayed diagnosis with hematuria, females also experienced delays in undergoing transurethral bladder resection compared to males. Additionally, studies have shown that the likelihood of receiving BCG therapy is similar for male and female NMIBC patients (Noon et al., 2013). For example, Jonathan et al. (Theodorescu et al., 2022) analyzed 472 (77.0%) male NMIBC patients and 141 (23.0%) female NMIBC patients who received BCG treatment and found no clear evidence of gender-based differences in treatment response, recurrence, and tolerability. Since these individuals did not have long-term BCG therapy or repeat transurethral resection, the generalizability of these findings is still questionable. It is worth noting that a population-based cancer registry study found that female NMIBC patients had significantly higher CSM than males (Noon et al., 2013). However, Alanee et al. (Alanee et al., 2015) analysis showed that females had a higher risk of CSM than carcinoma *in situ* (CIS) patients. In addition, female T1HG UCB patients had a greater chance of recurrence but not disease progression or death (Kluth et al., 2013). Similarly, no association between gender and illness development or recurrence was discovered in an analysis conducted by Boorjian et al. (Boorjian et al., 2010)of 756 male and 265 female patients undergoing BCG treatment. After examining 15,215 high-grade T1 patients, Martin-Doyle et al. (Martin-Doyle et al., 2015)found that while there was no association between females and cancer-specific survival or tumor recurrence, they did have a notably increased risk of disease progression. Konrad Bilski et al. (Bilski et al., 2022) conducted a retrospective analysis of 388 male and 131 female patients with primary high-risk NMIBC treated with transurethral resection (TUR) and found that females were associated with an increased risk of disease recurrence, but there was no gender difference in disease progression.

Although the reasons for the gender disparities in post-cystectomy death rates have not been fully elucidated, several researchers have provided evidence of inequalities in the quality of treatment received by male and female cystectomy patients. A retrospective study by Cárdenas-Turanzas et al. (Cárdenas-Turanzas et al., 2008) showed that female patients undergoing RC had significantly longer hospital stays and higher blood product costs. According to Siegrist et al. (Siegrist et al., 2010), female patients undergoing cystectomies experienced increased blood loss, longer operating times, more transfusion needs, and decreased pelvic lymph node dissection incidence. Additionally, an analysis of 12,722 UCB patients from the SEER database from 1988 to 2006 showed that female patients undergoing RC had a 20% higher risk of death within 90 days (Liberman et al., 2011b). However, other research has demonstrated the absence of significant gender disparities in frequently employed surgical quality measures, such as lymph node counts and surgical margin status (Horstmann et al., 2008; Kluth et al., 2014; Messer et al., 2014; Mitra et al., 2014). As a result, it is unlikely that the marginal differences in overall survival rates between men and women can be attributed simply to unequal treatment between the sexes. Future studies should determine the fundamental causes of gender-specific variations in diagnostic features and pathological staging (Table 1).

**TABLE 1:** Sex-specific differences in outcomes.

Aspect	Gender	Findings/Results
Bladder Cancer Incidence	Male	Diagnosed at a rate approximately three times higher than females (Shariat et al., 2010)
Bladder Cancer Mortality	Male	Likelihood of dying from bladder cancer is about twice that of females, resulting in a lower CSM-to-incidence ratio for males (Shariat et al., 2010)
Lifespan Reduction	Female	Female patients have a greater reduction in lifespan compared to males (6.5 years vs. 3.9 years) (Scosyrev et al., 2012)
Survival Differences	Female	Female individuals have a lower prognosis across all disease stages, including 5-year survival rates at different stages (Mungan et al., 2000a)
Registry Studies in Elderly Populations	Female	Female UCB patients in elderly populations have higher tumor staging and lower survival rates (Mungan et al., 2000a; Mungan et al., 2000b)
Japanese Kanagawa Cancer Registry Study	Female	CSM increased by approximately 40% in females, with higher cancer staging at initial diagnosis (Moschini et al., 2019)
Radical Cystectomy (RC)	Female	Limited data on gender differences in survival rates; some studies suggest female gender is an independent risk factor for CSM after RC (Tilki et al., 2010a; Tilki et al., 2010b; Kluth et al., 2014)
RC Series Studies on Muscle-Invasive BC	Female	5 out of 8 RC series studies reported higher CSM rates in female patients (Gago-Dominguez et al., 2001)
Early-Stage Prognostic Effects	Female	Some studies found gender-specific prognostic effects in early-stage disease, with lower survival rates in females (Kiemeney et al., 1997; Fliss et al., 2000)
Non-Metastatic Muscle-Invasive BC	Female	Female bladder CSM was poorer than that of males; females more likely to undergo RC with fewer complications (Cappellen et al., 1999)
Adjuvant Chemotherapy	Female	Female patients had higher CSM in a multivariate analysis (Cordes et al., 2013)
MIBC and NMIBC	Female	Female patients had poorer prognosis at particular stages, and obese females had poorer survival (Theodorescu et al., 2022)
T4 Bladder Tumors	Female	Female patients with pT4 bladder cancer had worse outcomes than males (Tilki et al., 2010c; Moschini et al., 2019)
SEER Database Analysis	Female	Higher independent risk of CSM in females with pT3–pT4 UCB (Liberman et al., 2011a)
NMIBC Patient Registry Study	Female	Female NMIBC patients had significantly higher CSM than males (Alanee et al., 2015)
Post-Cystectomy Disparities	Female	Disparities in treatment for female cystectomy patients, with longer hospital stays and higher blood product costs (Cárdenas-Turanzas et al., 2008; Siegrist et al., 2010)
SEER Database Analysis (Post-RC)	Female	Female patients undergoing RC had a 20% higher risk of death within 90 days (Liberman et al., 2011b).

### 5.2 Sex-specific responses in bladder cancer treatment

In addressing the efficacy of immune checkpoint inhibitors (ICIs) in bladder cancer treatment, it is imperative to explore potential sex-specific responses. Recent studies have suggested differential efficacy between male and female patients undergoing ICI treatment (Aragaki et al., 2022; Lindner et al., 2023). These investigations, characterized by variations in response rates and survival outcomes, underscore the importance of considering sex as a critical factor in immunotherapeutic interventions. Mechanisms underlying these sex-specific differences remain an active area of research, with hypotheses centered around hormonal, immunological, and genetic factors. Despite the promising strides made in systemic immunotherapy, challenges persist in the realm of bladder infusion chemotherapy, where localized drug delivery occurs directly into the bladder. Presently, bladder infusion chemotherapy lacks specific drug treatment options tailored to sex differences, necessitating further exploration and research. Ongoing initiatives are focused on identifying novel agents and optimizing drug delivery methods, urging future studies to delve into sex-specific responses to emerging treatments for a more personalized and effective approach.

Gender-related factors significantly influence various facets of bladder cancer drug therapy. Metabolic enzymes, particularly liver enzyme activity, exhibit gender-specific differences impacting chemotherapy drug metabolism and treatment outcomes. Hormonal influences, exemplified by estrogen and androgen receptor expression on bladder cancer cells, contribute to variations in hormone-targeted therapy responses. Studies indicate potential gender-related differences in immune checkpoint inhibitor responses, highlighting gender’s crucial role in immunotherapy outcomes. Gender-specific variations in body composition and distribution influence drug pharmacokinetics, impacting treatment effectiveness and toxicity. Additionally, psychosocial factors and gender-specific side effects influence patient experiences and compliance with bladder cancer drug therapy. Ensuring adequate gender representation in clinical trials is crucial for generalizability, particularly understanding interactions with hormone replacement therapy in postmenopausal women. Exploring the reasons behind gender-related survival disparities in bladder cancer guides efforts to tailor treatment strategies for improved outcomes.

Leveraging gender-specific characteristics for bladder cancer treatment involves a comprehensive approach to enhance therapeutic strategies and patient prognosis. Precision medicine, driven by genomic profiling, facilitates the development of targeted therapies tailored to individual profiles. Hormone-targeted therapies, modulating estrogen and androgen receptors based on expression patterns, offer gender-tailored interventions. Optimization of immunotherapy considers gender-related variations in immunological responses, ensuring enhanced treatment outcomes. Gender-specific pharmacokinetics in drug development ensure individualized dosing, optimizing drug exposure and efficacy. Inclusive clinical trial designs with adequate gender representation generate robust data to discern gender-specific responses to emerging therapies. Tailored psychosocial support programs address unique coping mechanisms and adherence challenges, contributing to overall wellbeing during treatment. Gender-tailored screening protocols, survivorship programs, and educational initiatives empower healthcare providers and communities to enhance early detection, survivorship care, and awareness of gender-related risk factors, advancing personalized, equitable, and effective bladder cancer therapies.

## 6 Conclusion

In conclusion, our comprehensive exploration of bladder cancer underscores the profound impact of sex on its multifaceted dimensions, illuminating crucial insights for future research and clinical interventions. The etiological panorama, encompassing factors such as smoking, occupational exposures, and genetic mutations, exhibits intriguing disparities in male and female populations. The nuanced biological differences, including the intricate interplay of sex hormones, sex chromosomes, metabolic enzymes, and the microbiome, provide a rich substrate for understanding the intricacies of bladder cancer initiation and progression.

Furthermore, our synthesis of gender-specific diagnostic and prognostic implications reveals substantial variations across all disease stages, transcending the detection phase and implicating fundamental disparities in disease outcomes. The dearth of conclusive evidence regarding gender-specific survival rates post-radical cystectomy calls for further exploration and validation. Addressing these disparities necessitates tailored research initiatives and the development of gender-specific treatment modalities.

In essence, this comprehensive review serves as a clarion call for heightened attention to sex-specific considerations in bladder cancer research and clinical practice. A more nuanced understanding of these gender-based disparities is essential for advancing personalized medicine and optimizing outcomes for all patients afflicted by this prevalent malignancy. Future investigations should delve deeper into the intricate interplay of sex-related factors, fostering a more precise and equitable approach to bladder cancer prevention, diagnosis, and treatment.

## Figures and Tables

**Table:** 

**BC**	Bladder Cancer
**HRA**	High-Risk HPV (Human Papillomavirus)-Associated
**KRAS2**	Kirsten Rat Sarcoma Viral Oncogene Homolog 2
**RB1**	Retinoblastoma 1
**FGFR3**	Fibroblast Growth Factor Receptor 3
**STAG2**	Stromal Antigen 2
**TERT**	Telomerase Reverse Transcriptase
**ESPL1**	Extra Spindle Pole Bodies Like 1
**UTX**	Ubiquitously Transcribed Tetratricopeptide Repeat X-Linked
**MLL**	Mixed-Lineage Leukemia
**CREBBP**	CREB-Binding Protein
**EP300**	E1A Binding Protein P300
**NCOR1**	Nuclear Receptor Corepressor 1
**ARID1A**	AT-Rich Interaction Domain 1A
**PTEN**	Phosphatase and Tensin Homolog
**MIBC**	Muscle-Invasive Bladder Cancer
**AR**	Androgen Receptor
**BCG**	Bacillus Calmette-Guérin
**CD**	Cluster of Differentiation
**ER**	Estrogen Receptor
**GSTM1**	Glutathione-S-transferase M1
**H3K27me3**	Histone H3 Lysine 27 Trimethylation
**KDM6A**	Lysine-specific Demethylase 6A
**MIBC**	Muscle-Invasive Bladder Cancer
**UCB**	Urothelial Carcinoma of the Bladder
**UGT**	UDP-glucuronosyltransferase
**MIBC**	Muscle-Invasive Bladder Cancer
**UCB**	Urothelial Carcinoma of the Bladder
**SEER**	Surveillance, Epidemiology, and End Results
**CSM**	Cancer-Specific Mortality
**HR**	Hazard Ratio
**NMIBC**	Non-Muscle-Invasive Bladder Cancer
**CIS**	Carcinoma *In Situ*
**TUR**	Transurethral Resection
